# Tailored Screening for Late-Life Depression: A Short Version of the Teate Depression Inventory (TDI-E)

**DOI:** 10.3389/fpsyg.2019.02693

**Published:** 2019-12-05

**Authors:** Michela Balsamo, Aristide Saggino, Leonardo Carlucci

**Affiliations:** School of Medicine and Health Sciences, Università degli Studi G. d’Annunzio Chieti e Pescara, Chieti, Italy

**Keywords:** depression, elderly, late-life, adults, Rasch analysis, item response theory

## Abstract

A number of assessment instruments have been developed as efficacy measures of geriatric depression in clinical trials but most showed several weaknesses, such as time-consuming administration, development and validation in younger populations, and lack of discrimination between anxiety and depression. Among the extant self-report measures of depression, the 21-item Teate Depression Inventory (TDI; Balsamo and Saggino, 2013), developed *via* Rasch analysis, showed a satisfactory level of diagnostic accuracy, and allowed the reduction of false positives in test scoring in adult population. The present study explored the potential improvement in the psychometric performance of the TDI in the elderly by item refinement through Rasch analysis in a sample of 836 elderly people (49.5% males; mean age = 73.28; SD = 6.56). A resulting shorter version was composed of the best-fitting and discriminative nine items from the full form. The Teate Depression Inventory (TDI-E) (E for elderly) presented good internal construct validity, with unidimensional structure, local dependency, good reliability (person separation index and Cronbach’s alpha), and no signs of differential item functioning or measurement bias due to gender and age (65 vs. 75+ years). Cut-off points and normative data provided could enhance the clinical usefulness of the TDI-E, which seems to be a promising valid and reliable tool for the screening of geriatric depression, with less risk of finding false positives due to overlapping of depression in elderly with other comorbid conditions.

## Introduction

### Depression in Elderly and Its Measurement

Among older adults, depression is a common with more persistent and debilitating consequences than other forms of psychological distress condition (Arean and Ayalon, 2005; Friedman et al., 2007; Gilchrist and Gunn, 2007; Rodda et al., 2011; Sözeri-Varma, 2012). Among these, diminished cognitive, physical, and social functioning, increasing of risk of morbidity, general self-neglect, dependence by the others and mortality are those mainly noteworthy (Unützer et al., 2000, 2002; Fiske et al., 2009; Grover and Malhotra, 2015; Kennedy et al., 2016).

Late-life depression is characterized by different ways of presentation with respect to depression earlier in the lifespan (Koenig et al., 1993). Elderly depressed people are more likely to be affected by concomitant medical illness and psychiatric problems that can complicate their detection and therapy. For example, the presence of somatic and dementing disorders, the comorbidity with anxiety and physical complaints, may be misattributed to depression or vice versa (Lyness et al., 1995; Beekman et al., 2000; Friedman et al., 2007; Gilchrist and Gunn, 2007; Sözeri-Varma, 2012; Kennedy et al., 2016). For 516 depressed patients aged 70 years and older, suffered from a concomitant medical illness (e.g., weight loss, somatic anxiety, middle insomnia, and work impairment) eight items of the Hamilton Rating Scale for Depression (HAMD, Hamilton, 1960) may be elevated by the concurrent somatic disorder (Linden et al., 1995). Thus, the detection and assessment of elderly depression could be overlooked, misunderstood, or even misattributed because its symptoms can be easily confused with those of medical problems (e.g., fatigue, loss of involvement, pleasure, and interest in sexual activity, trouble sleeping, appetite, or weight change) and/or with natural cognitive functioning decline, including problems of concentration and memory, and/or with the senescence, an irreversible decline in mental and physical capabilities, as well as with some anxiety symptoms, including the hypochondriasis (Clark and Watson, 1991; Lyness et al., 1995). Ideally, depression assessment should be restricted to items that avoid confounding by medicall illness.

Lastly, items tapping pessimism, reduced actvity or interest, thoughts of death, possible suicidal intention, and meaning of the life have a different meaning for those approaching the end of their life, compared to younger individuals (Cusin et al., 2009). Probably, problems of unique interpretation could only be addressed if an experienced interviewer administers the scale of depression, but this turns out to be the case (Balsamo et al., 2018).

Given its costly and wide-ranging implications and the different psychopathological expression, sound and specific measurement of late-life depression is mandatory to improve the recognition and treatment of depressed elderly patients.

### Current Self-Report Instruments on Geriatric Depression

A number of self-report measures developed in the adult population have been used to assess the incidence and intensity of depression symptoms and to monitor anti-depressant treatment progresses in the elderly (Andersen, 1999). Indeed, despite the differences in depressive symptoms between adult and geriatric population, the primary outcome measures used for the antidepressant trials in the people aged 65 years or older are still the self-report instruments developed in the adult population, such as the Beck Depression Inventory-II (BDI-II; Beck et al., 1996), the Center for Epidemiological Studies Depression Scale (CES-D, Radloff, 1977), and the HAM-D (Bent-Hansen et al., 2003; Roose et al., 2004; Sheikh et al., 2004; Wohlreich et al., 2004).

Nevertheless, controversies are emerged about their psychometric quality in the elderly because of some relevant shortcomings, such as time-consuming administration, vulnerability to misinterpretation and response biases, questionable structure of their response formats, and dependence of their scores on cultural factors (Balsamo and Saggino, 2007). For example, according to methodological studies, the number of items could be shortened by about 70% without compromising the measurement properties substantially (Moran et al., 2001). For the extant depression scales, it was highlighted that short forms with as few as nine items performed in ways very similar to the full version, while a version composed of only five items had a detectable difference from the full version (Cheung et al., 2007). Shorter form of the extant depression scales currently used for elderly should permit to decrease the overall time testing, in order to reduce the survey fatigue or boredom that older participants may feel, mostly when taking longer measures made up of many similar or repetitive items (Balsamo et al., 2018).

Moreover, some of these scales vary in terms of their primary content focus and their coverage of the core symptoms of depressive symptomatology. This aspect, which cast doubt on their content validity, could result in the under-recognition of depressive symptoms (Faravelli et al., 1986; Balsamo and Saggino, 2007).

Among the scales designed with the specific aim of screening depression in the elderly, the 30-item GDS was the gold standard (Yesavage et al., 1982). However, it has been repeatedly criticized (e.g., Friedman et al., 2005), mainly because of its length (Jongenelis et al., 2005; Chachamovich et al., 2010). The 15-item GDS-SF, extracted by the full-length form based on the base of diagnostic accuracy criteria (Yesavage and Sheikh, 1986), was also criticized (Chiang et al., 2009; Mitchell et al., 2010a,b; Wongpakaran et al., 2019) because of its lacking unidimensional nature. Indeed, two- and three-factor models emerged in different samples of elderly (Incalzi et al., 2003; Brown et al., 2007). Moreover, several items (i.e., #2, #3, and #10) were found to have a low clinical validity since did not contribute to the construct of geriatric depression and to be more related to subjective aspects of depression (e.g., life satisfaction or cognitive impairment) (Tang et al., 2005; Chiang et al., 2009; Wongpakaran et al., 2019). Further, some daunting multidimensional issues, such as differential item function (DIF), item misfit and redundancy, have been highlighted through IRT approach (Tang et al., 2005; Chachamovich et al., 2010; Wongpakaran et al., 2019). The development of the GDS brief forms (GDS-10, -6, -5, -4, and -1; Mitchell et al., 2010a,b) raised supplementary problems, including the difficulty to compare scores across different cultures and languages. In addition, the forced binary (yes or no) response format potentially provides no indication about the relative intensity or frequency of depression symptoms experienced by elderly (Castle and Engberg, 2004). Thus, to avoid these unidimensional and diagnostic problems, the GDS-SF is usually included together with other methods of screening for depression in a wide range clinical assessment for geriatric sample (Chiang et al., 2009).

Summing up, a brief, specific and unidimensional method of assessment of the severity of depressive symptoms in older adults seems to be the answer to the main challenges posed by the measurement of depression in this population (Balsamo et al., 2018). The different presentation of the depressive psychopathology between adults and elderly imposes different and specific measures in these populations. Measures specifically designed to measure depression in older adults result to lack of unidimensionality, i.e., an important requirement for calculating and interpreting a total score of an instrument (Lichtenberg, 2010; Ziegler and Hagemann, 2015). As a result, special emphasis should be laid on the investigation of the unidimensional structure of the scales used in the elderly general population.

Additionally, in most epidemiological studies, more females than males were diagnosed with depression (Albert, 2015), although these reported rates might be due to the use of generic diagnostic criteria and psychometric instruments that are not sensitive to depression in men (Oliffe and Phillips, 2008). As regards, age, there is some concern that older adults can obtain inflated scores on self-report depression instruments, which stem from non-depressive sources (e.g., medical problems) (e.g., Joiner et al., 2005). About this, it is worth noting that there is a difference between young- and old-old subjects groups (Garfein and Herzog, 1995; Mehta et al., 2008). Therefore, a further open question remains whether *bias-free* dimensional assessment of depression, independent of age, somatic morbidity, and gender, is feasible in the elderly general population.

Rasch measurement model is a powerful modern approach to develop unidimensional and bias-free instruments in health sciences. It examines both the scale and individual item performance in depth, leading to measures of depression, which are sample free and item free, and without DIF due to gender and age (Embretson and Reise, 2000). To our knowledge, no Rasch-based self-report measure of geriatric depression was developed. Up to the present, few IRT models have been applied only in the shortening process of extant few measures of depression used in the elderly, developed within classical test theory (CTT). The deriving advantage was to provide an improvement in the psychometric performance by item refinement, e.g., by revealing item redundancy, so that these instruments could be shortened without information loss (Tang et al., 2005; Lamoureux et al., 2009; Chachamovich et al., 2010; Forkmann et al., 2013; Spangenberg et al., 2015).

### The Teate Depression Inventory

Among the extant self-report measures of depression used in older people, the 21-item Teate Depression Inventory (TDI; Balsamo and Saggino, 2013) was developed within Rasch logistic approach of responses. The TDI had shown to have an excellent Person Separation Index (PSI), no bias due to item-trait interaction, and control of major response sets (Innamorati et al., 2013, 2014; Balsamo et al., 2013a,b, 2014, 2015a,b,c, 2016, 2019; Saggino et al., 2017; Carlucci et al., 2018a,b). Three cut-off scores were recommended in terms of sensitivity, specificity, and classification accuracy for screening for varying levels (minimal, mild, moderate, and severe) of depression severity in a group of patients diagnosed with major depressive disorder (Balsamo and Saggino, 2014). More recently, applying the Bayes’ theorem, the TDI showed to allow significant reduction of false positives in test scoring in clinical and non-clinical samples (Tommasi et al., 2018). Indeed, it was found to overcome the 50% level of diagnostic accuracy, unlike the BDI, the HAMD, the Zung Self-Rating Depression Scale (ZSDS; Zung, 1965), and the CES-D, because of a good procedure to select test items and subjects with clearly defined pathological symptoms.

About the pitfalls in the measurement of the geriatric-specific characteristics of late-life depression, the TDI significantly related to measures of anxiety and depression in expected directions and showed promise discriminating depression from anxiety (Picconi et al., 2018a,b). As such, it displayed significantly (*p* < 0.01) higher correlation with depression measure (GDS) compared with the anxiety measure, both trait and state, both cognitive and somatic scales, in a sample of 396 community-dwelling middle aged and elderly adults (Balsamo et al., 2015b).

Regarding the sex, the performance of the TDI has been found to be sufficiently insensitive for gender biases in a sample of 529 subjects (229 psychiatric outpatients and 300 healthy community-dwelling adults). Indeed, all items showed no difference due to gender, except for the item #10. It could represent an advantage over the extant depression questionnaires (like the BDI-II), that included several items showing DIF dependent of the respondent’s sex since they might substantially interfere with the valid interpretation of instrument’s sum score (Santor et al., 1994; Forkmann et al., 2009; da Rocha et al., 2013).

Regarding the impact of the somatic multimorbidity on the measurement of depression, the TDI was a unidimensional screening instrument of depression that included no items referring to somatic complaints (sleep and appetite disturbances). Present in an initial set of items, they did not fit the Rasch model because of no additional information provided to estimate the person’s depression level. The lack of these items results to be consistent with the confounding of comorbidity that may be expected when applied to other diagnostic groups and can result in false positives (Thombs et al., 2007; Gibbons et al., 2011; da Rocha et al., 2013), as well as more useful for assessing depression in somatically ill patients, as are most of the elderly. Indeed, total scores of existing depression scales containing somatic items could be biased if those were filled from patients suffering from somatic illnesses because they did not reflect depression severity.

Although these compelling psychometric characteristics, the length of the TDI could be a limitation, which hinders its widespread use in elderly population. Reading and filling out its 21 items can be stressful for some older respondents, as well as not very useful for practitioners interested in measuring multiple constructs or repeated measurement of constructs, in the presence of time constraints. Moreover, even if the TDI is a Rasch-based measure, it is preferable to verify its psychometric functioning in a special population, like that of the elderly. Indeed, although Rasch analysis specifies that item parameters be sample free, constant item parameters imply a constant construct while different item parameters across samples of the relevant population could imply that the construct has changed, as Linacre (1996) outlined. Depressive psychopathology among elderly patients has been shown to be different in some aspects from younger individuals. Thus, given the construct of depression could change in different populations, it is desirable to test the TDI performance in the elderly population, which is different from the adult population, for which the TDI was developed.

Another point worth nothing concerns the availability of age-relevant norms in assessing mental health disorders among older adults (Therrien and Hunsley, 2012). Specific cut-off point represents a point of demarcation along continuum to address clinical decision and to identify good candidates for psychological treatments or protocols by clinicians interested to routinely screen their older patients for depression. This is particularly useful in clinical research, where the number of patients who receive the same intervention is usually limited. Only some depression measures currently used for measuring geriatric depression cut off points were computed. As regards the norms, few self-report instruments showed adequate normative data for elderly, which limited their clinical value (Breeman et al., 2015). With the growing number of older adults who is requiring mental health services, the diagnosis and treatment selection is helped by assessment data; thus, it is mandatory to have measures that are normed for an older population (Edelstein et al., 2007).

### The Present Study

The present study aims at shortening and adapting the TDI to the elderly population using Rasch analysis with special emphasis on its unidimensional structure and DIF due to gender and age. Adherence of the brief TDI for elderly to Rasch model assumptions was determined with the analysis of Rasch model and item fit, unidimensionality, local dependency (LD) (principal component factor analysis of the residuals and correlation matrix of residuals), reliability (PSI and Cronbach’ alpha), and DIF with regard to participants’ age (65 vs. 75+ years) and gender.

A secondary aim was to examine the choice of cut-point to identify older people as depressed for screening and diagnostic purposes.

Finally, norm values were calculated. Based on the individual raw sum scores, each person’s latent trait score *θ* was calculated and transformed linearly into percentiles, *z* values (mean = 0; SD = 1) and *t* values (mean = 50; SD = 10).

## Methods

### Participants and Procedure

The sample included 836 elderly participants, of whom 49.5% were males. They were, on average, 73.28 (SD =6.56) years. Included in the sample was a subsample of 80 elderly clinical depressed participants (69% males) with an average of 72.60 years (SD = 5.44) years. No statistical differences were found in age variable between clinical vs. nonclinical group (*t*_834_ = −1.207, *p* = 0.304). Non-clinical participants have been enrolled by licensed psychologists at various community centers; groups; associations, senior citizens’ Universities in Central and Southern Italy. They were preliminarily screened for psychiatric illness with a short interview. Only individuals evidencing no current psychopathology, no history of psychiatric hospitalization, and no cognitive impairment or neurological diseases (e.g., dementia, Parkinson, and Alzheimer’s disease) were included in the non-clinical sample. Depressed participants were extracted from the standardization sample (Balsamo and Saggino, 2013). They were recruited from mental health counseling services and from private centers by clinical psychologists and psychotherapists. Eligible depressed participants were screened for major depressive disorders using the Structured Clinical Interview for the Diagnostic and Statistical Manual of Mental Disorders Axis I (SCID-I; First et al., 1997). Only participants who met the Diagnostic and Statistical Manual of Mental Disorders criteria (5th ed.; DSM–5; American Psychiatric Association, 2013) for a primary diagnosis of depression were included in the clinical subsample.

The study was reviewed and approved by the Department of Psychological Sciences, Health and Territory, University of Chieti, Italy, Review Board. All our procedures were in accordance with the ethical standards of the 1964 Declaration of Helsinki and its later amendments. Written informed consent was obtained from all individual participants included in the study.

### Measure

#### Teate Depression Inventory

The Teate Depression Inventory (TDI) was composed of 21 items that aimed to assess symptoms of major depression during the past 2 weeks (Balsamo and Saggino, 2013). Participants responded on a five-point Likert scale ranging from “never” to “always.” Total scores were created by first reverse-coding several items and then summing single items. Higher total scores indicate more severe depressive symptoms.

### Data Analysis

The analysis plan consisted of two consecutive steps: initial evaluation of unidimensionality of the TDI using Mokken analysis and evaluation of Rasch model assumptions.

Firstly, a Mokken analysis was carried out within the framework of IRT in order to assess the assumption of unidimensionality. Following Sijtsma et al. (2011) recommendations, unidimensionality for polytomous-item measures was investigated through the Automated Item Selection Procedure (AISP) algorithm developed in *Mokken p*ackage of R, using recommended value of *c* = 0.3 and *α* = 0.05 (Molenaar and Sijtsma, 2000). The AISP algorithm aimed at partition a set of items (or a set of unscalable items) into Mokken scales (Mokken, 1971; Sijtsma and Molenaar, 2002). Mokken scale is defined by a set of dichotomously scored items for which, given a lower bound “*c*,” all inter-item covariances are positive and scalability coefficients (*H*_i_/*H*) were set ≥ *c* > 0. This definition can be extended to polytomous scored items. To date, values of 0.3 < *H*_i_/*H* < 0.4 identified weak scalability; values of 0.4 < *H*_i_/*H* < 0.5 as moderate, and values of *H*_i_/*H* > 0.5 as strong scalability (Mokken, 1971).

Next, in line with the previous literature on the TDI (Balsamo and Saggino, 2013; Balsamo et al., 2014), data were fitted to the Rasch measurement model using RUMM2030 (Andrich et al., 2010). According to the Rasch model, probability of a person endorsing a dichotomic item was a logistic function of the difference between the person’s abilities and the item difficulty (Rasch, 1960). Because the Rasch model was originally developed for intelligence and attainment tests, the “item difficulty” (Rasch, 1960) can be “translated” as and the severity of depression expressed by the item, i.e., the probability (expressed in logits) to endorse a high category of an item: for “difficult” items this probability would be lower than for “easy” items, relative to the individual person measure. Similarly, person’s abilities are referred to as the latent trait score or person measure. If the Rasch model holds, persons and items can be scaled along a single linear latent continuum (i.e., depression). Since the TDI was conceived as a polytomous scales, an extended parameterization of the Rasch model for dichotomous responses (the Rating Scale Model, RSM) was used to fit the logistic function between the severity of depression and the severity of depression expressed by the items (Andiel, 1995). Like the Rasch model, the RSM and others extended models for polytomous scales (i.e., the Partial Credit Model) can be categorized as generalized linear model (GLM), with random effects modeling for the subject ability (Raju et al., 2014).

Data were found to fit Rasch model when the observed patterns of response are close to the expected model and satisfy a series of assumptions: local independency, response category ordering, lack of item bias or DIF, overall model and individual item fit, and reliability. Rasch analysis represents an iterative process where an initial observed pattern of response was tailored to ensure the overall fit of the data to the model. In this view, a series of sequential steps and fit indices has been estimated. In details:

1. assumption of stochastic ordering of the items along the whole latent trait was determined by a series of fit statistics within adequate ranges (Andrich, 1988): (1) chi-squared statistics (*χ*^2^) and probability ($\chi_{\mathrm{prob}}^{2}$) should be not significant at level *α* = 0.05 with Bonferroni adjustment (also named item-trait interaction); (2) items with fit residual values >|2.5| (95% CI) should be discharged from the model; (3) summary item and person fit residual statistics should be approximated to the normal *z* distribution with mean = 0 and SD = |1| (or approximately |1.4|);
2. monotonicity for polytomous items was assessed by the inspection of the ordered items category thresholds. Thresholds represent the transition point between categories. When ordered, the amount of the probability of the category response itself leads to an amount of the latent trait (i.e., depression);
3. assumption of local response independency was assessed performing a Principal Component Factor Analysis of the Residuals (PCFAR; Smith and Miao, 1994; Linacre, 1998). Local independence implies that when controlling for the main Rasch dimension, no high or substantial residual correlations between the items shall remain. Hence, high residual correlation values (higher than the absolute value > 0.2; Marais, 2013) revealed that performance on the items was accounted for by a third trait dimension (Lee, 2004; Baghaei, 2008), displaying LD issue or multidimensionality. In addition, LD inflated reliability and affected parameters estimation (Wright, 1996);
4. DIF for age (60–75 years/over 75) and gender (males/females) person factors was also evaluated for each item by the two-way ANOVA (*α* = 0.05 with Bonferroni adjustment). DIF or item bias may occur systematically in responses based on characteristics of the respondents (*trait*) (uniform DIF) and varying along the construct (non-uniform DIF). In this study both, the uniform and non-uniform DIF issues were assessed;
5. afterward, strict unidimensionality was tested on the shortened set of items using the Smith’ test of unidimensionality implemented in the RUMM 2030. A series of independent *t* test was performed in order to compare person estimates from two sets of items, composed, respectively, of items with positive and negative factor loadings (*λ* ≥ |0.30|) on the first principal components analysis of the residuals (Smith, 2002). If more than 5% of these *t* tests was found to be significant, the resulting scale was labeled as multidimensional;
6. reliability and scale targeting were evaluated in order to assess the measurement validity of the final model. Reliability has been evaluated using the PSI. Values of PSI from 0.70 to 0.85 identified the minimum requirement for group and individual person measurement; a PSI > 0.85 was considered excellent (Nunnally, 1978). In addition, the internal consistency of the scale was examined by Cronbach’s α. Targeting was measured by comparing graphically the mean location score obtained for the participants with that of the items: good values should be located in the center of the scale, close to the zero. Targeting of the person-item threshold distribution assesses how well individual item difficulties and individual person abilities can be matched on a common logit scale (Andrich, 1988) and how are the ceiling and floor effects (Tennant et al., 2004).

Next, following Davis et al. (2008), a regression analysis was performed to determine how well the resulting Rasch interval scale predicted the TDI scores, as conventionally computed using Likert interval scale (e.g., the raw summed scores of all the items), by fitting a cubic model.

To facilitate the clinical use of the TDI short version, norms values were computed. Person’s latent trait scores (*θ*, expressed in logits) were transformed to an interval-metric scale using the original TDI 0–4 range scores (Tennant and Conaghan, 2007; Lundgren-Nilsson et al., 2013). This transformation is allowed since “Rasch model is capable of constructing linear measures from counts of qualitatively ordered observations, provided the structure of quantity is present in the data” (Salzberger, 2010). Next, the trait scores (*θ*) were transformed linearly into percentiles, *z* and *t* values.

Further, the receiver operating characteristic (ROC) curve analysis (Gleitman, 1986) with the Youden index (*J*) method was employed in order to detect the cut-off score, potentially useful in determining clinically depressed elderly. In this case, the optimal cut-off score represents the *J* function of the difference between true positive rate and false positive rate over all possible cut-point values. In the present sample, the prevalence rate of depression was 10.4% (*N* = 80). The performance of a diagnostic variable was quantified by computing the area under the curve (AUC; Bradley, 1997). Optimal values of AUC ranged from 0 “weak performance” to 1 “perfect performance” (Hanley and McNeil, 1982), with a value of >0.70 as recommended (Swets et al., 2000).

## Results

### Mokken Analysis

After rescoring those formulated in reversed mode, all the TDI items were submitted to Mokken analysis, in order to test the unidimensionality assumption. The AISP revealed that all the TDI items loaded on a single latent dimension. The inter-item covariances were found positive, thus satisfied the first criterion of the Mokken scale. Next, all the item scalability coefficients (*H*_i_) ranged from 0.350 (weak) to 0.470 (moderate); hence, the second criterion of a Mokken scale has been satisfied. The scalability coefficient for the entire TDI scale (*H*), equals to 0.409, showed a moderate scalability. Then, the assumption of unidimensionality was met for the 21 items of the TDI.

### Rasch Analysis

The initial Rasch model was run with all the 21 items of the TDI exhibiting, an excellent PSI of 0.91. No floor and ceiling effects have been found. However, the initial model showed a poor overall model fit [*χ*^2^ = 309.57(189), *p* < 0.001], and four items displayed disordered thresholds. The mean fit residual was 0.773 (SD = 2.066), indicating that the items did not fit the model properly, with an observed modest local response dependency. Out of the 21 items, six exhibited misfit criteria, including large fit residuals (±2.5) and significant *χ*^2^ probabilities (*p* < 0.0001) with Bonferroni adjustment.

Since our goal was to develop a brief measure of depression for elderly people, attempts were made to improve fit to the initial model, by collapsing categories to achieve sequential order in items with disordered thresholds. The remaining items showed properly ordered thresholds, and all response categories were retained.

After collapsing item thresholds and ordering categories, the results showed non-considerable change (see Model #2 in Table 1). Thus, shortening of the TDI has been continued toward a final model, using an iterative strategy. Firstly, LD was pursued by deleting the pairs of items with correlations exceeding 0.3 were taken to indicate dependency. Items misfitting were removing item-by-item if displayed fit residuals outside the acceptable range (±2.5) and/or *χ*^2^ probability value of the individual item fit was significant. Lastly, item bias or DIF for age and gender was also evaluated to determine if it was contributing to the misfit of items.

**Table 1:** Summary fit statistics for Rasch analyses.

Model	# Items	Items				Persons				Item-trait interaction		PSI
		Location		Fit residual		Location		Fit residual				
		Mean	SD	Mean	SD	Mean	SD	Mean	SD	*χ*^2^	*χ*^2^ prob	
Initial	21	0.000	0.389	0.772	2.066	−8.836	0.969	−0.402	1.783	309.57	0.0000	0.91
1	21	0.000	0.425	0.539	1.880	−8.741	1.019	−0.403	1.767	315.68	0.0000	0.91
Final	9	0.000	0.264	0.410	0.959	−0.714	0.969	−0.411	1.386	97.53	0.1017	0.83

*PSI, pearson separation index (person/item)*.

After removing item by item all misfitting items by the 21-item set, best model fit (with Bonferroni adjustment) was achieved by a final nine-item set, named the Teate Depression Inventory (TDI-E) (E for elderly) (Table 2). The final solution showed good fit to model expectations, with a not significant item-trait interaction index [*χ*^2^ = 97.53(81), *p = 0*.101]. Its item mean was 0.00 and SD = 0.264. No local response dependency was observed within the nine-item TDI model, as revealed by the inspection of the PCA residual correlations matrix. All item thresholds were found ordered, excepting for item #6. For achieving its sequential order, the “rarely” and “sometimes” response categories were collapsed. An inspection of the category response frequencies revealed that elderly participants chosen these two categories with the same probability (rarely = 14.64%; sometimes = 15.24%).

**Table 2:** Final model with nine items.

Item/content	DSM diagnostic criterion	Location	SE	FitResid	*χ*^2^	*χ*^2^ prob	*F*-stat	*p*
TDI1\feeling blue	VII	−0.465	0.041	0.50	12.892	0.1676	1.630	0.1024
TDI13\fatigability	VI	−0.063	0.037	0.246	8.952	0.4417	1.073	0.3804
TDI18\loss of interest	II	−0.056	0.036	−0.519	9.978	0.3522	1.148	0.3259
TDI8\concentration ability^*^	VIII	−0.044	0.041	1.756	5.904	0.7495	0.592	0.8041
TDI15\enjoyment^*^	I	−0.025	0.04	0.998	6.086	0.7313	0.653	0.7520
TDI11\loss of self-confidence	VII	−0.009	0.039	1.561	12.395	0.1919	1.588	0.1145
TDI2\concentration difficulty	VIII	0.000	0.039	−0.92	12.854	0.1693	1.644	0.0987
TDI14\lack of energy^*^	VI	0.089	0.04	0.722	8.826	0.4535	0.94	0.4895
TDI6\withdrawal	IX	0.572	0.06	−0.647	19.643	0.0202	2.534	0.0072

*χ^2^ prob with Bonferroni adj. = 0.0055; DSM, diagnostic and statistical manual of mental disorders*.

* *Reverse scored items*.

There was no DIF for both gender or age, based on Bonferroni adjusted *p*’s. Strict unidimensionality test (Smith, 2002) performed on the TDI-E showed that only the 5% (CI: 3.5–6.5%) of the paired *t* tests fell outside the 95% confidence interval, hence the assumption of unidimensionality held. The PSI of 0.83 indicated an adequate person separation reliability (Andrich, 1982) and also suggested that the power to detect items that do not fit the model was good. The TDI-E also showed high internal consistency (Cronbach’s *α* = 0.85).

**Figure 1:**
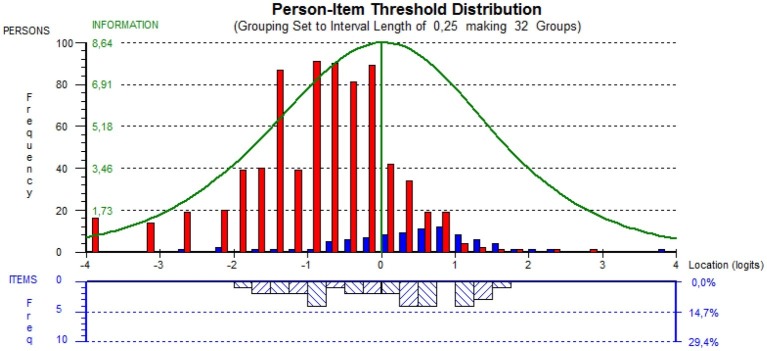
Targeting of person and item. Red bars, non-clinical; blue bars, clinical.

The shortened scale displayed an unbalanced person-item targeting to the left side of the person threshold distribution plot (easier questions or greater severity of depression to endorse the item), with a percentage of extreme scores <5%. No floor and ceiling effects have been found. However, the TDI-E was well targeted to the clinical sample, with the means of the person being 0.435 (SD = 1.01) on the logit scale (Figure 1).

Given the drastic scale reduction of the TDI (leading from 21 to 9 items), it was evaluated how the Rasch scale predicted the summed score of the selected nine items. Results from regression analysis supported the appropriateness of the cubic function into predicted Rasch-based scores in relation to the summed score. Summary and coefficient estimates for the raw scores were displayed in Table 3.

**Table 3:** Logits scores regressed by raw summed score for the nine items model.

	Model estimates			
	Coefficient	SE	*t*	*p*
Constant	−3.4184	0.0159	−214.815	<0.0001
(Raw summed score)^3^– cubic trend	0.0003	0.0001	51.448	<0.0001
(Raw summed score)^2^– quadratic trend	−0.0176	0.0003	−58.630	<0.0001
Raw summed score – linear trend	0.4026	0.0041	98.605	<0.0001
	**Model fit**		
	*R*	*R*^2^	Adjusted *R*^2^	
	0.995	0.989	0.989	

Next, since Rasch model conformity of the TDI-E has been confirmed, norm values were determined. As no DIFs for gender and age groups were found, the score of the TDI-E resulted to be independent from gender and age. Norms values were displayed in Table 4. Rasch-based scores for all the raw summed score shave been estimated by transforming the Person’s latent trait scores (*θ*) to their interval scale equivalent scores (or Rasch interval scale). This transformation is valid if no missing value was observed in the TDI items. Practically, a raw summed score of 10 (*θ* = −0.86) on the TDI-E is equivalent to a Rasch interval score of 1.58, with a *Z* value of −0.49 (31st percentile) and a *T* score of 45.

**Table 4:** Transformation of raw score to Rasch-based scores.

Raw scores	*θ*	Rasch interval scale (0–4)	Z	%	*T*
0	−3.90	0.00	−2.27	1	27
1	−3.11	0.41	−1.81	4	32
2	−2.57	0.69	−1.49	7	35
3	−2.21	0.88	−1.28	10	37
4	−1.93	1.03	−1.11	13	39
5	−1.69	1.15	−0.97	17	40
6	−1.49	1.25	−0.86	20	41
7	−1.31	1.35	−0.75	23	43
8	−1.15	1.43	−0.66	26	43
9	−1.00	1.51	−0.57	29	44
10	−0.86	1.58	−0.49	31	45
11	−0.73	1.65	−0.41	34	46
12	−0.60	1.72	−0.33	37	47
13	−0.48	1.78	−0.26	40	47
14	−0.36	1.84	−0.19	42	48
15	−0.25	1.90	−0.13	45	49
16	−0.13	1.96	−0.06	48	49
17	−0.02	2.02	0.01	50	50
18	0.09	2.08	0.07	53	51
19	0.21	2.14	0.14	56	51
20	0.32	2.20	0.21	58	52
21	0.44	2.26	0.28	61	53
22	0.56	2.32	0.35	64	53
23	0.68	2.38	0.42	66	54
24	0.81	2.45	0.50	69	55
25	0.94	2.52	0.57	72	56
26	1.09	2.59	0.66	75	57
27	1.24	2.67	0.75	77	57
28	1.41	2.76	0.85	80	58
29	1.61	2.87	0.97	83	60
30	1.83	2.98	1.10	86	61
31	2.11	3.13	1.26	90	63
32	2.47	3.31	1.47	93	65
33	3.00	3.59	1.78	96	68
≥34	3.79	4.00	2.25	99	72

*θ, estimated Pearson’s latent trait score for depression; %, percentiles; *Z* (*M* = 0, SD = 1); *T* (*M* = 50, SD = 10)*.

### Receiver Operating Characteristic Curve Analysis

A ROC curve analysis was performed to compare the non-depressed elderly group vs. the depressed group. Results indicated that the nine-item TDI scale was able to discriminate the two groups being examined. In details, the optimal cut-off point useful for the screening and diagnostic purposes was detected. The AUC for the TDI-E total score was 0.833 (95% CI of 0.806–0.858), suggesting good discrimination between the groups. The Youden index (0.54, CI 0.42–0.62) for the TDI-E total score was observed at a score of 18 points, corresponding to a sensitivity of 69% and specificity of 85%. Positive and negative predictive power were 35.5 and 96%, respectively, and overall diagnostic efficiency was 84%. Alternative cut-off values (see Table 5), were also estimated *via* BCa bootstrapped 95% CI (Efron and Tibshirani, 1993; Zhou et al., 2002). For instance, a cut-off of >11 could be employed for the screening purpose, corresponding to a sensitivity of 90.8% and specificity of 57.3%. Positive and negative predictive powers were 18.4 and 98%.

**Table 5:** Alternative cut-off values for the TDI-E.

Cut-off	Youden	Sensitivity	95% CI	Specificity	95% CI	+LR	95% CI	−LR	95% CI	+PV	95% CI	−PV	95% CI
>6	0.204	95.4	88.6–98.7	24.97	21.9–28.2	1.27	1.2–1.4	0.18	0.07–0.5	12.9	12.2–13.6	97.9	94.7–99.2
>11	0.441	90.8	82.7–95.9	53.27	49.6–56.9	1.94	1.8–2.2	0.17	0.09–0.3	18.4	16.9–20.0	98	96.3–99.0
>13	0.471	82.76	73.2–90.0	64.35	60.8–67.8	2.32	2.0–2.7	0.27	0.2–0.4	21.2	19.1–23,6	97	95.3–98.1
**>18**	**0.544**	**68.97**	**58.1–78.5**	**85.45**	**82.7–87.9**	**4.74**	**3.8–5.9**	**0.36**	**0.3–0.5**	**35.5**	**30.6–40.8**	**96**	**94.5***–***97.0**
>20	0.518	62.07	51.0–72.3	89.72	87.3–91.8	6.04	4.6–7.9	0.42	0.3–0.6	41.2	34.9–47.8	95.3	94.0–96.4
>23	0.387	43.68	33.1–54.7	95.06	93.3–96.5	8.84	6.0–13.1	0.59	0.5–0.7	50.7	40.9–60.4	93.6	92.3–94.6
>25	0.267	28.74	19.5–39.4	98.00	96.7–98.9	14.35	7.9–26.2	0.73	0.6–0.8	62.5	47.8–75.2	92.2	91.2–93.1

*In bold, the recommended cut-off value*.

*+LR, positive likelihood ratio; −LR, negative likelihood ratio; +PV, positive predictive values; −PV, negative predictive values*.

## Discussion

An appropriate answer to the several issues posed by challenges to measurement of late-life depression could reside in a self-reported measurement late-life depression, with the characteristics of brevity, specificity, and unidimensionality (Balsamo et al., 2018).

Concerning the brevity, it is well known that brief tools in primary care would be very useful for general practitioners, who are scarce of time and their high frequent patients may be elderly (Luber et al., 2001; Frank et al., 2018).

Several briefer versions of the GDS, the gold standard measure for depression in the elderly, have been developed. However, they have not been shown to be exempt from weakness. For example, in a meta-analytic study on their diagnostic accuracy, there was inconsistency in the items that contributed to these briefer versions and there are no standardized cut-off scores. This cast doubt on the validity of their scores, as well as on their diagnostic performance (Pocklington et al., 2016).

Concerning the unidimensionality, extant scales currently used in the elderly general population has been found lacking because some items related to a different latent trait, such as physical illness, were included (Osman et al., 2004; Storch et al., 2004; Crockett et al., 2005). As a result, using a single total score could result in its unclear interpretation. For example, two patients with the same summed score might differ in terms of the relative severity and frequency of different components of depression; thus, a treatment targeting only one of these aspects would be harder to detect in its effect. By applying the Rasch analysis, it is possible to develop unidimensional and *bias-free* measures of depression in the elderly general population.

The TDI is a newly developed Rasch-based measure of depression. Given the necessity of brevity of measurement in older adults, Rasch analysis was employed to develop a briefer measure of geriatric depression from the Rasch-based 21-item TDI. Given the differences in depressive symptoms between geriatric and adult populations, this study aimed at evaluating its performance in this specific population.

### Mokken and Rasch Analyses

In line with the previous literature, Mokken analysis of the TDI items showed that they mapped on to the depression trait, with medium scalability coefficients. To select items from the 21-item TDI with best measurement properties for composing a briefer, homogeneous, and unidimensional scale of geriatric depression, a Rasch analysis was performed. A shortened measure with nine items was derived. The newly developed TDI-E included items covering a wide range of diagnostic criteria of the DSM-5 for the major depressive episode (for a comprehensive review of the criteria, see Balsamo et al., 2014). Like the TDI, the TDI-E covered the same patterns of difficulties, within the range ± 1 logit. Item #1 (“I felt down”) resulted to be the easiest to endorse, while item #6 (“I felt the desire to retire and disappear”) was found the most difficult to endorse. This result was in accordance with previous literature (e.g., Lewinsohn et al., 2003), according to which depressed mood is the common symptom of depression, more so than anhedonia and other symptoms. Similarly, wish to die was considered a component of suicidal desire, an extremely important indicator of dangerousness across categories of mental disorders, including depression (Joiner et al., 2005). All the TDI-E items displayed no significant differences in the thresholds distances, suggesting that respondents discriminated properly between response options. Only item #6 showed two collapsed categories to achieve sequential order. As suggested by Bode (1997), ambivalent categories in rating scale (e.g., “do not know”) often share more noise than information and should be threatened as missing data, so the pivot point for collapsing categories may be in the middle of uncertain categories. Notably, elderly respondents with reduced working memory capacity were more prone to answer “do not know” or to choose ambivalent categories in difficult questions, compared to respondents with higher cognitive abilities (Knäuper et al., 1997, 2016).

Like the TDI, the TDI-E demonstrated no DIF with regard to participants’ age (65 vs. 75+ years) and gender. This means that all the TDI-E items performed equivalently for males and females, and for young old and old-old subjects (Garfein and Herzog, 1995; Mehta et al., 2008).

Prior evidence demonstrated that females showed an elevated risk of major depressive episode, and this risk increase in elderly females (+65 years) (Angst et al., 2002; Kessler et al., 2010). Potentially, this unbiased version of the TDI could allow an easy and efficient assessment of depression among elderly, thus avoiding the extensive use of differentiated norms (e.g., by gender or age) that are complex and may be difficult to communicate to general audiences or within a multidisciplinary team of experts.

The present study supported unidimensional construct of geriatric depression of the TDI-E. As revealed by the strict test of unidimensionality, neither subset of item from the factorial analysis of the residuals showed a significant difference on person estimates from the nine-item measure. Reliability, as measured by the PSI, was 0.83, an acceptable level especially for individual level data, which indicated not too large reduction from the PSI of 21-item TDI (0.96). A significant reduction of PSI values in short self-report measures derived from long self-report measures was expected (Davis et al., 2008; Shea et al., 2009). Unlike coefficient Cronbach’s alpha, the PSI was not affected (or inflated) by the test length (Mallinson et al., 2004). Nevertheless, a limited and homogenous range of items, e.g., items with a close range of abilities, potentially resulted in decreasing of variability detected or in an increasing the amount of error, leading to a decrease in reliability (Mallinson et al., 2004). The reliability issue could represent a limitation for the present study, since a small set of items has been selected from a homogenous sample of participants (mostly healthy), which potentially weakens the ability of the scale to differentiate people.

### Teate Depression Inventory Cut-off Scores and Diagnostic Utility

Results from regression analysis also revealed the measurement precision of the TDI-E. The raw summed scores for the nine items of the TDI-E seemed to predict the Rasch-based scores expressed in logits and the appropriateness of the cubic function (Lin et al., 2019). In other words, there is a substantial equivalence on the precision of the TDI-E score as measure of depression, whether it is computed as raw summed score or as Rasch-based interval score.

The diagnostic performance of the TDI-E in detecting elderly people who meet clinical thresholds for depressive symptoms, analyzed by the ROC curves, identified the cut-off point of 18 for differentiating non-depressed and depressed respondents. This value could facilitate researchers and clinicians into maximizing the clinical utility of the TDI-E when using in an applied way. For example, in clinical setting, a cut-off score easily allows to differentiate potential cases of clinical depression (True Positive) from probable “non-cases” (False Positive) or make decisions about who to treat and what treatments to provide (Widiger and Samuel, 2005; Van Dam et al., 2013). However, for clinicians who use the TDI-E as a screening instrument in clinical settings, where a higher sensitivity may be required, sensitivities and specificities corresponding to alternative cut off points were provided (Table 5).

Finally, although it may very tempting, to use a cut-off score on a self-report inventory as the single means of deriving, a diagnosis is a practice that should be avoided (Nezu et al., 2000). Rather, respondents scoring above the established cut-off level should be interviewed to assess for the depressive disorders criteria found in the DSM5.

### Teate Depression Inventory Norms

The presented normative data could offer important advancements for the interpretation of the self-report measure scores and enhance its usefulness for clinical and research applications. For example, the *z* and *t* scores, set out here, makes it possible to compare TDI-E scores with the distribution of summed scores arising from convergent/divergent measures of depression and anxiety (e.g., the GDS or the Geriatric Anxiety Inventory), both in the clinical and general population. Thus, researchers and clinicians could benefit from these data in order to estimate significant changes across treatment (especially in repeated assessment) and/or to perform a brief assessment of the patient’s depression severity. Moreover, the norms table provided makes the TDI-E scores comparable to the scores derived from other geriatric measures, even developed within the CTT.

### Limitations

These results were based in a convenience sample almost exclusively composed of healthy and cognitive intact older people. They may not might be different in a depressed and/or cognitive impaired older population. Another limitation raises from the choice to use the Rasch model to shorten the TDI. Within the IRT models, analysis of Rasch is a fairly straightforward model and showed advantages and limitations. One limitation concerns the Rasch assumption of equal measurement error for each item (no discrimination parameters were provided, like in the 2PL model), as well as the possibility that a simple model may not fit the data. However, as Ryan outlined (Ryan, 1983), the inclusion of adjunctive parameters, i.e., the discrimination or guessing parameters (in the 2PL and 3PL models, respectively) could make potentially difficult and ambiguous the interpretation of item difficulties because all parameters are estimates simultaneously (Andrich, 2004, 2011; Han, 2012). Far from others IRT models, the Rasch model estimated a single person and item parameters; thus, the total score represents a sufficient statistic for the person parameter (Andrich and Marais, 2019). Further limitation concerns the lack of the investigation on test-retest reliability of this instrument and on the correlations with external measures for assessing its concurrent and discriminant validity.

Future investigations will be devoted (1) to verify if it displays validity coefficients with well-known depression and anxiety questionnaires currently used in the elderly; (2) to define its responsiveness to different contexts and different clinical samples (i.e., elderly with cognitive impairment or dementia); and (3) to examine if the TDI-E is composed of cultural-invariant items, which could then be applied in transcultural investigations free of bias.

The TDI has been translated in English and Portuguese, in order to be used as an outcome measure in internationally based longitudinal studies and clinical trials.

## Conclusion

The present study explored the potential improvement in the psychometric performance of the 21-item TDI in the elderly by item refinement *via* Rasch analysis. This resulted in a short version of nine items, which was unidimensional, showed good internal construct validity, good reliability, and no signs of DIF due to gender and age. A specific cut-off point provided here could be more meaningful for screening purpose, as well as its normative data. To sum up, the TDI-E seems to be a valid and reliable tool for the screening of geriatric depression, with less risk of finding false positives due to overlapping of depression in elderly with other comorbid conditions. Its brevity could improve feasibility and compliance of older adults, mostly when several self-report measures are being used in a multidimensional psychological assessment in late life.

## Data Availability Statement

The datasets generated for this study are available on request to the corresponding author.

## Ethics Statement

The study was reviewed and approved by the Department of Psychological Sciences, Health and Territory, University of Chieti, Italy, Review Board. Data were collected during 2018-2019. All our procedures were in accordance with the ethical standards of the 1964 Declaration of Helsinki and its later amendments. Written informed consent was obtained from all individual participants included in the study.

## Author Contributions

MB and LC designed the study. LC conducted the statistical analyses. MB, AS, and LC interpreted the data. MB and LC drafted the manuscript. All authors contributed toward drafting and revising the paper and agreed to be accountable for all aspects of the work.

### Conflict of Interest

We declare a potential conflict of interests for some authors, those who have published the handbook for one of the tests analyzed in the present report (AS and MB–please see “References” section).

The remaining author declares that the research was conducted in the absence of any commercial or financial relationships that could be construed as a potential conflict of interest.
